# Structural changes in the COPD lung and related heterogeneity

**DOI:** 10.1371/journal.pone.0177969

**Published:** 2017-05-25

**Authors:** Dana Copot, Robin De Keyser, Eric Derom, Clara Ionescu

**Affiliations:** 1 Department of Electrical energy, Systems and Automation, Ghent University, Technologiepark 914, 9052, Ghent, Belgium; 2 Ghent University Hospital, Department of Respiratory Diseases, De Pintelaan 185, 7K12, 9000, Ghent, Belgium; Medical University of Graz, AUSTRIA

## Abstract

This paper proposes a mathematical framework for understanding how the structural changes in the COPD lung reflect in model parameters. The core of the analysis is a correlation between the heterogeneity in the lung as COPD degree changes (GOLD II, III and IV) and the nonlinearity index evaluated using the forced oscillation technique. A low frequency evaluation of respiratory impedance models and nonlinearity degree is performed since changes in tissue mechanics are related to viscoelastic properties. Simulation analysis of our model indicates a good correlation to expected changes in heterogeneity and nonlinear effects. A total of 43 COPD diagnosed patients are evaluated, distributed as GOLD II (18), GOLD III (15) and GOLD IV (10). Experimental data supports the claims and indicate that the proposed model and index for nonlinearity is well-suited to capture COPD structural changes.

## 1 Introduction

Fractional calculus (FC) penetrated well the biomedical engineering field of research and numerous literature reviews witness its success [1, 2]. It has been shown that both time domain and frequency domain analysis can be performed with various FC tools. Already several decades ago, modelling lung mechanics have been subject to fervent applications of constant-phase element models, a successful tool emerging from fractional calculus [3–5]. Special attention has been given to viscoelastic properties, intrinsically requiring such models and providing relevant clinical information upon changes in lungs with disease [6–8].

Geometry of the lungs, either fractal or heterogeneous, associated with CT scans, has been employed to analyse images of lung tissue [9]. Since single fractal models of image processing algorithms for clinical analysis have been poor in capturing the high degree of variability in airway density, multi-fractal tools have been employed [9]. Same FC tools have been employed for image processing of other biological tissues as well [9, 10].

In our previous work, we employed structure and mechanical properties to develop consistent impedance models for respiratory tree [11–13]. We have shown that such models converge to lumped impedance models with fractional order terms to account for frequency dependent dynamics [13, 14]. We expected that changes from airway remodelling with disease affecting morphology and structure would lead to changes in model parameters; this hypothesis has been verified in both simulations as well as in clinical studies.

Changes in chronic obstructive pulmonary disease (COPD) are affecting mainly distal airways and lung parenchyma, involving mechanisms related to small airway disease [15]. Alveolar structure and morphology is drastically affected and thus plays an important role in determining the viscoelastic properties of lung parenchyma. Consequently, the diffusion mechanism is greatly dependent on these changes.

The goal of our study is to analyse what are the effects induced by these changes at alveolar levels upon impedance model parameters. We expect that since the structure plays a role in determining the appearance of a fractional order term in the impedance, this term may change as a result of changes due to disease. The core of the paper is to evaluate if there is a correlation between the heterogeneity in the lungs (as COPD degree progresses) and the non-linear index.

The paper is organized as follows: In Section II the theoretical background and the proposed theoretical framework is presented. In this section the case of one alveolus and the case of a network of alveoli is discussed. Next, the structural changes in patients with chronic obstructive disease are presented, as well as a short description of the device used to acquire the data. In section III the simulation and experimental results are shown and discussed followed by the conclusions.

## 2 Materials and methods

### 2.1 Theoretical background

In the context of electrical analogy, one usually considers voltage as the equivalent for respiratory pressure (*P*) and current as the equivalent for airflow (*Q*). The pressure in function of time is the accumulation of these components with the trans-pulmonary pressure at end-expiration (*P*_0_):

$$\begin{array}{c} P\left(t\right)=\frac{1}{C}V\left(t\right)+R\frac{dV}{dt}+I\frac{d^{2}V}{dt^{2}}+P_{0} \end{array}$$ (1)

where elastance (1/*C*) (the reciprocal of the compliance) is directly related to volume (*V*), the resistance (*R*) is related to airflow and the inertia (*I*) is related to accelerating particles. This equation represents the global relationship between airflow and pressure in the lungs. Its equivalent frequency domain form is the respiratory impedance as a function of oscillatory frequency [16].

This principle has been applied by means of Womersley theory and Navier-Stokes equations to elastic tubes preserving structure and morphology of the airways [11]. According to the seminal work of Weibel and Mandelbrot [17–19], the structure of the lungs can be roughly approximated by a recurrent dichotomous tree of 24 levels, leading to a rough approximation of 2^24^ alveolar ducts. At the end of each alveolar duct there is a porous bag called alveoli, which are grouped together like a lot of interlinked caves, rather than individual sacks.

A mathematical model of the respiratory tree up to 24th level has been proposed [11] and used in subsequent works in clinical studies [13]. The model is based on the geometrical parameters: radius and wall thickness (*r*, *h*), on the mechanical characteristics of the airway tube: complex elastic moduli (given by its modulus (|*E*|) and angle(*φ*_*E*_)) on the Poisson coefficient (*ν*_*P*_), and on the air properties: viscosity and density (*μ*, *ρ*). Over the length *ℓ* of an airway tube, we have the corresponding definitions for electrical resistance:

$$\begin{array}{c} R_{e}=\ell \frac{\mu \delta^{2}}{\pi r^{4}M_{1}}\sin\left(\varepsilon_{1}\right), \end{array}$$ (2)

and for electrical capacitance

$$\begin{array}{c} C_{e}=\ell \frac{2\pi r^{3}{\left(1-\nu_{P}^{2}\right)}^{2}}{\left|E\right|h}\cos\varphi_{E}, \end{array}$$ (3)

where, *M*_1_ is the modulus of the Bessel function of order 1, *ε*_1_ is the phase angle of the complex Bessel function of order 1, and $\delta =\sqrt{\omega \rho /\mu }$ the Womersley parameter.

We have shown that the geometry of the lungs leads to a piecewise fractal structure, whose electrical equivalent leads to a recurrent ladder network. The admittance of such network has the form:

$$\begin{array}{c} Y_{N}\left(s\right)≅\frac{1/R_{e1}}{1+\frac{1/R_{e1}C_{e1}s}{1+\frac{1/\lambda R_{e1}C_{e1}s}{\ldots \frac{\ldots }{1+\frac{1/\chi^{N-2}\lambda^{N-1}R_{e1}C_{e1}s}{1+1/\chi^{N-1}\lambda^{N-1}R_{e1}C_{e1}s}}}}} \end{array}$$ (4)

with *R*_*e*1_ and *C*_*e*1_ denoting the resistance and compliance in the first airway (i.e. trachea), and λ the ratio of resistances per total levels, *χ* the ratio of compliances per total levels and *s* the Laplace operator. This continuous fraction expansion can be well-approximated for *N* → ∞ airway levels by the compact form of admittance [20]:

$$\begin{array}{c} Y_{N}\left(s\right)≅\frac{1/R_{e1}}{K\left(\lambda ,\chi \right)\cdot {\left(1/R_{e1}C_{e1}s\right)}^{n}} \end{array}$$ (5)

with *K*(λ, *χ*) a gain factor depending on the values of the ratios and the fractional order *n* given by

$$\begin{array}{c} n=\frac{\log(\lambda )}{\log(\lambda )+\log(\chi )} \end{array}$$ (6)

Notice that the impedance, *Z*_*N*_(*s*) will be the inverse of the admittance. In the frequency domain, the fractional order will lead to a constant-phase behaviour, i.e. a phase-locking in the frequency range given by the convergence conditions [13, 20]. The lumped version of this model is the well-known constant-phase element model given by:

$$\begin{array}{c} Z_{CP}\left(s_{k}\right)=R+I\left(s_{k}\right)+\frac{1}{C(s_{k}^{\beta })} \end{array}$$ (7)

where *R* (kPa s/L) and *I* (kPa s^2^/L) denote the central airway resistance and inertance respectively, whereas the last term consists of a constant-phase element which can be split into a real and imaginary part, denoting tissue damping and tissue elastance. These two latter properties are defined as averaged over the frequency range evaluated by the lung function test [3–5].

$$\begin{array}{c} \begin{array}{c} G_{r}=\frac{1}{C\omega_{k}^{\beta }}\cos\left(\beta \pi /2\right)\\ H_{r}=\frac{1}{C\omega_{k}^{\beta }}\sin\left(\beta \pi /2\right)\\ \eta_{r}=\frac{G_{r}}{H_{r}} \end{array} \end{array}$$ (8)

and their ratio denotes the degree of heterogeneity present in the tissue [21]. This ratio is in fact a characterization of sources for nonlinear contributions in the respiratory dynamical properties. If changes in *R* are small, then any changes in *G*_*r*_ (kPa s^(1−*β*)^/L) will represent changes in parenchyma or in very small airways. The memory effects are introduced via *s*^(1−*β*)^ [22]. These effects are observed in materials with viscoelastic properties. Chronic changes in *H*_*r*_ (kPa s^(1−*β*)^/L) reflect changes in the intrinsic mechanical properties of the parenchyma [23]. This parametric model has been shown to reliably estimate airway and tissue properties [24], and its sensitivity to bronchodilatation in dogs at low frequencies has been assessed in [3]. The same model has been used to assess respiratory impedance in rats at low frequencies in [25]. The model has been used to evaluate respiratory impedance in healthy and sick patients [13, 14, 21]. The reliability of the parameter estimates from model fitting to raw data has shown that the inertance *I* and the resistance *R* may contain a high degree of uncertainty, while the constant-phase term delivers a reliable estimate of peripheral tissue properties. The study also showed that the steep dependency on frequency at low frequencies in the real part of impedance is consistently fitted by the constant-phase model from Eq (7) when compared to other models from literature.

In order to quantify the non-linear contributions the following index has been introduced [13]:

$$\begin{array}{c} T=\frac{P_{even}+P_{odd}}{P_{exc}}\cdot \frac{U_{exc}}{U_{even}+U_{odd}} \end{array}$$ (9)

where each variable represents the sum of the absolute values of all the contributions in the pressure and flow signals a non-excited frequencies (even and odd) and the odd excited frequencies. The index expresses the relative ration of the contributions at non-excited frequencies with respect to the contributions at the excited frequencies. For a detailed overview on filtering techniques applied to extract the contributions see [13, 26].

### 2.2 Proposed theoretical framework

Work of breathing in the lungs takes place with loss of kinetic energy and heat loss (production of heat through friction of air in airways and airductuli). To allow a systematic approach, we need to use an impedance model of one alveolus which will be later used for analysis of alveolar sac structures and their respective properties in presence of remodelling effects.

#### 2.2.1 Impedance of one alveolus

Let us assume a spherical alveolus characterized by an inner radius *r*_1_ and an outer radius *r*_2_. We may assume without loss of generality that heat conduction is anisotropic, heat diffusion is unidirectional and spherical coordinates can be used for modelling. Following the line of thought presented in [22, 27], approximating the spherical wall (i.e. difference between inner radius and outer radius) with many cells whose pressure is governed by:

$$\begin{array}{c} \frac{dP_{i}}{dt}=\frac{1}{R_{i}C_{i}}\left(P_{i-1}-2P_{i}+P_{i+1}\right) \end{array}$$ (10)

with *R*_*i*_ and *C*_*i*_ representing the resistance and compliance properties of the cell *i* and *P*_*i*_ pressure profile assumed uniformly distributed along the cell face. The thickness of each cell is then given by the relation: *h* = *r*_2_ − *r*_1_/*N*, with N the total number of cells and 0 ≤ *i* ≤ *N*. Introducing *N*_0_ = *r*_1_/*h*, it follows that the radii of each cell is given by *r*_*i*_ = (*N*_0_ + *i*)*h*. The surface of the cell becomes:

$$\begin{array}{c} S_{i}=4\pi r_{i}^{2}=4\pi h^{2}{\left(N_{0}+i\right)}^{2} \end{array}$$ (11)

and it follows that the resistance of each cell is given by:

$$\begin{array}{c} R_{i}=\frac{h}{\lambda \cdot S_{i}}=\frac{1}{4\pi \lambda h{\left(N_{0}+i\right)}^{2}} \end{array}$$ (12)

where λ is the thermal conductivity (W m^−1^ °C^−1^). The capacity per cell is defined as:

$$\begin{array}{c} C_{i}=\frac{4}{3}\pi h^{3}\left[3{\left(N_{0}+i\right)}^{2}+\frac{1}{4}\right]\rho c \end{array}$$ (13)

with *ρ* mass density (kg m^−3^) and *c* specific heat (J kg^−1^ °C^−1^). The ladder network of series connected cells with resistance and capacity calculated in Eqs (12) and (13) in Laplace operator, provides the explicit impedance which can be evaluated over a range of frequencies. This can be either a ladder network made of recurrent elements, either one with same element values per cell. In our case, the latter applies since we assumed a homogeneous density of the alveolar wall. The lumped transfer impedance with heat loss of such a ladder network of this spherical alveolus is given by:

$$\begin{array}{c} \lim_{s⟶\infty }Z_{s}\left(s\right)=\frac{\sqrt{a}}{\lambda \left(4\pi r_{1}^{2}\right)s^{0.5}} \end{array}$$ (14)

with *s* the Laplace operator, *a* = λ(*ρ* ⋅ *c*) thermal diffusivity (m^2^ s^−1^). For specific values of lung tissue and frequencies (*s* → *jω*) one may evaluate this lumped impedance. This lumped impedance has an operator at non-integer order, implying a constant phase of 45 degrees.

#### 2.2.2 Network of alveoli

**Fig 1:**
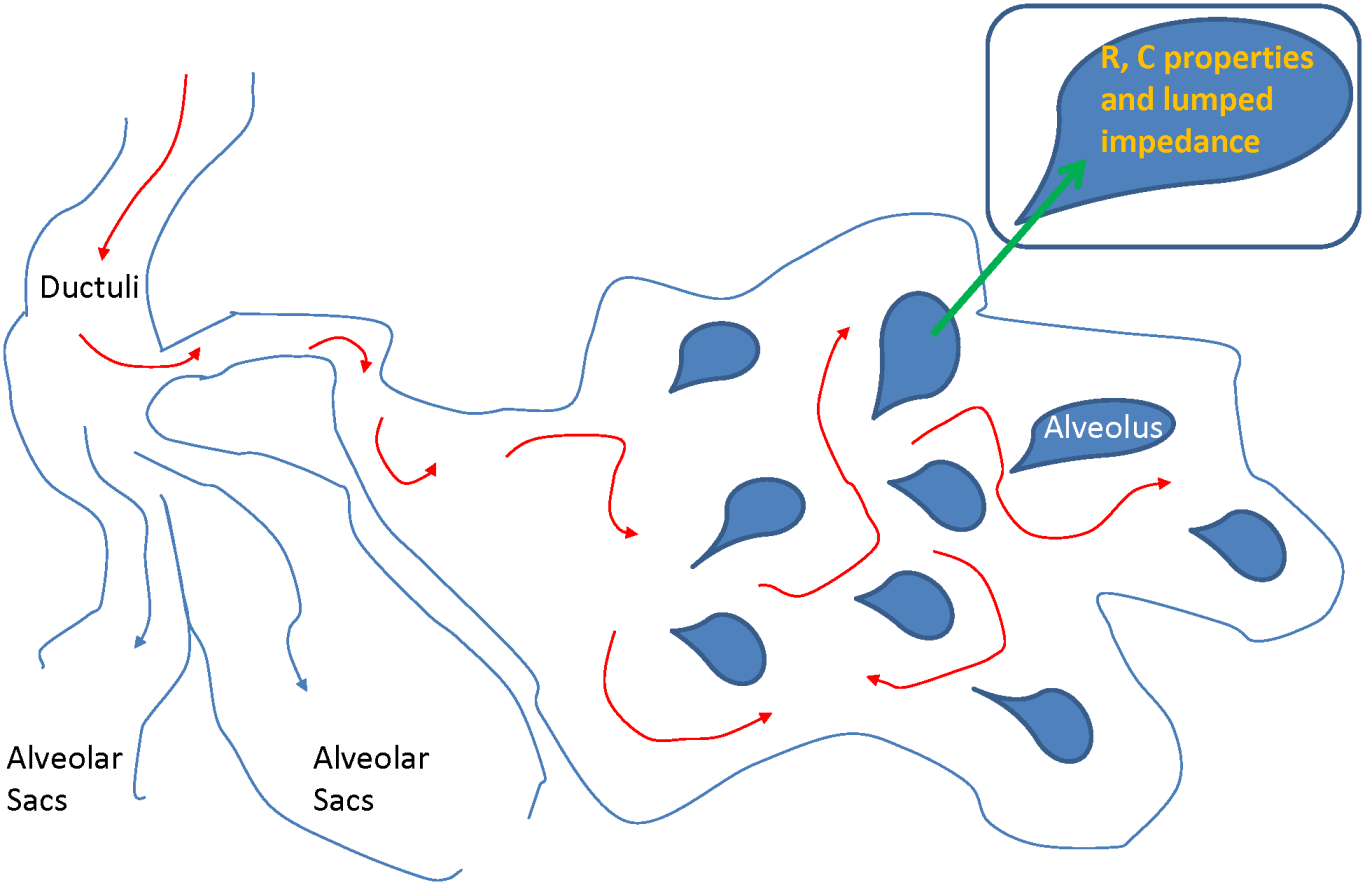
Conceptual view of air passing through a network of alveoli. In this picture, the airflow has to reach every alveolus characterized by its own resistance, capacitance and resulting lumped impedance.

Air in the lungs passes terminal bronchi, enters respiratory bronchi and consequently reaches the alveolar ducts and alveoli. This may be interpreted as penetration of air through a porous material, i.e. lung tissue, whose density increases with distance. Fig 1 illustrates the aforementioned transport phenomenon. The material is assumed to be homogeneous, sponge-like, whereas every alveolus has same properties. This assumption corresponds to healthy lung tissue.

Fractal properties have been applied to intergranullar materials for impedance models and sphere like geometry has been employed successfully to proof convergence to a fractional order impedance model [28, 29]. The structure of such porous materials has been modelled with FC tools and led to fractal dimensions between dimension 2 (surface) and 3 (volumetric). Depending on the density of the material, the values for fractal dimension changed accordingly. In the context of lung pathology, changes in density occur in emphysematous lungs. The fractional order impedance term values reflects these changes and thus we speculate it might be of interest to study this aspect.

**Fig 2:**
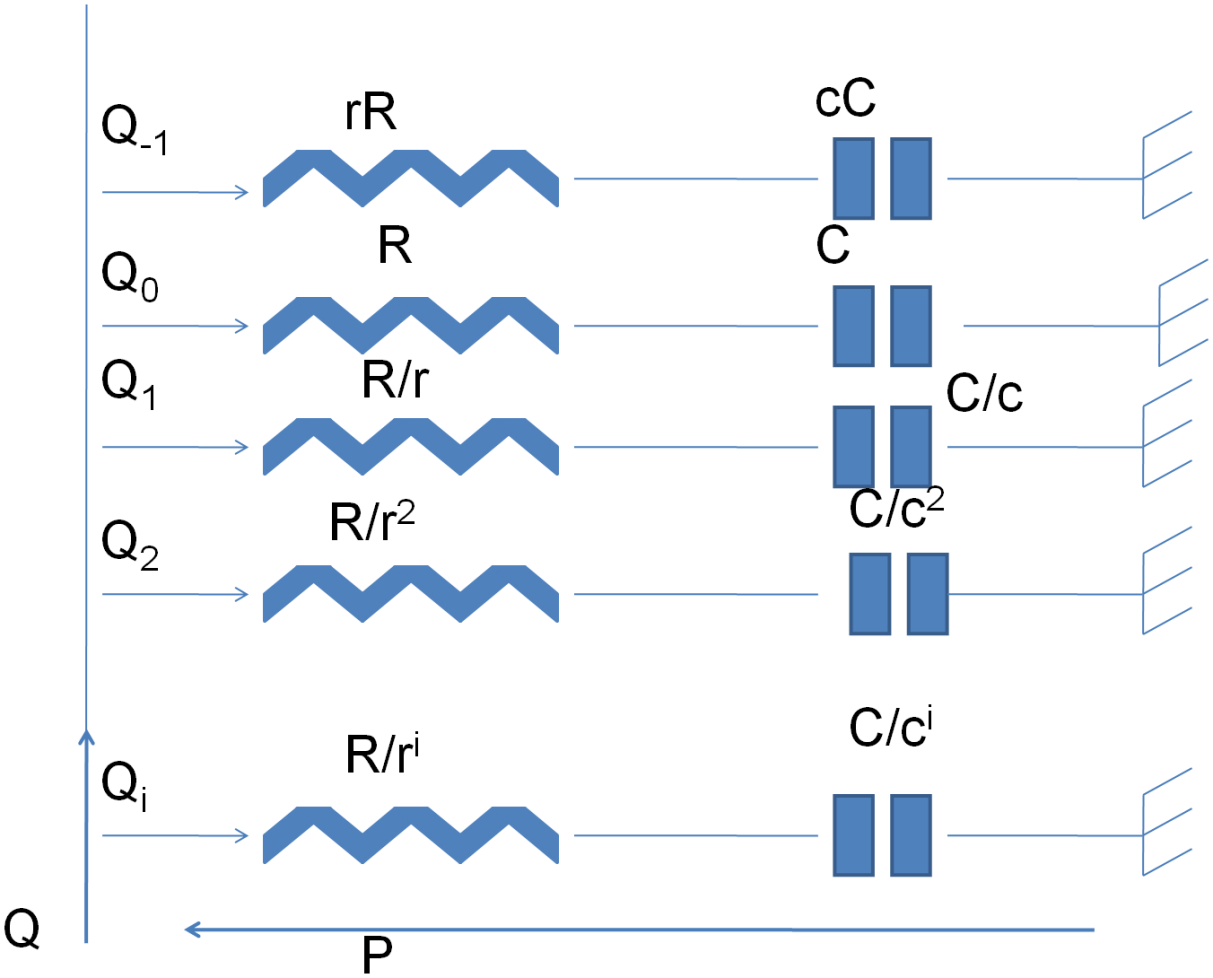
Recursive parallel arrangement of series RC cells. The variables *r* and *c* are recursive ratios for resistance and capacitance, respectively.

To illustrate the effect of changes in structure in tissue lungs, we will first discuss the nominal conditions of healthy lung tissue. The tools we employ are similar as for one alveolus, equivalent ladder network models. Porous material is not necessary permeable, therefore the pores we consider in our model are not linked to each other, thus similar to alveolar sacs (dead-end). To enter these pores, the airflow must counteract resistance with dissipative energy and must inflate/deflate with an elastic capacitance which decreases the peak pressure for the same given volume of air. The flow entering from the main stem we assume equally divided between the alveolar pores. Mandelbrot introduced the fractal character to porosity because of self-similarity principle [19]. In this context, we may employ recurrent ladder networks of parallel cells—each cell denoting an alveolus. Although Mandelbrot introduced fractality in porous materials as randomly distributed (si ze and location), due to the very compact structure of the lung ductuli we may consider this parallel arrangement of cells as deterministic [17]. Fig 2 depicts the recursive parallel arrangement of series RC cells.

In an idealized context, fundamental law of physics for air mass is given by:

$$\begin{array}{c} M\cdot v(t)\cdot S+F(t)=0 \end{array}$$ (15)

with *v* the air velocity, *M* the air mass and *F* the force. Air flow is given by *Q*(*t*) = *v*(*t*) ⋅ *S*, with *S* the alveolar surface. Air pressure is then introduced as *P*(*t*) = *F*(*t*)/*S*. Notice that in case of disease, the breathing surface is decreased, and the same amount of pressure will require a higher force—which is indeed the case of increased work of breathing in patient with various degrees of respiratory obstruction.

The arrangement from Fig 2 has an admittance of the form:

$$\begin{array}{c} Y\left(s\right)=s\sum_{i}\frac{C_{i}}{1+C_{i}R_{i}s} \end{array}$$ (16)

with $C_{i}=\frac{C_{i}+1}{c}$ and $R_{i}=\frac{R_{i}+1}{r}$ with their recurrent ratios. We have shown previously that such ladder networks converge to a lumped form [13, 30]:

$$\begin{array}{c} Y\left(s\right)≃{\left(\frac{s}{\omega }\right)}^{m} \end{array}$$ (17)

with $m=\frac{r}{c+r}$ the fractional order term value dependent exclusively on the ratios of the alveolar parameters. This relation defines the admittance of the air-alveolar interface.

Assuming initial conditions zero for both air flow and pressure at the interface (deviation values with respect to operating conditions), then Eq (15) becomes:

$$\begin{array}{c} \frac{M}{S^{2}}\frac{dQ(t)}{dt}+P\left(t\right)=0 \end{array}$$ (18)

and in Laplace transform:

$$\begin{array}{c} \frac{M}{S^{2}}\left[sQ\left(s\right)-Q_{0}\right]+P\left(s\right)=0 \end{array}$$ (19)

and

$$\begin{array}{c} Q\left(s\right)=\frac{1}{\omega_{0}^{n}}s^{n}P\left(s\right) \end{array}$$ (20)

which has similar form as the last term in Eq (7). Combining these relations leads to:

$$\begin{array}{c} Q\left(s\right)=\frac{\tau^{n}s^{n}}{1+\tau^{n}s^{n}}\frac{Q_{0}}{s} \end{array}$$ (21)

where *n* = 1 + *m* with *m* (from Eq (17)) the non-integer order differentiator and $\tau ={\left(\frac{M}{S^{2}}\frac{1}{\omega_{0}^{m}}\right)}^{\frac{1}{n}}$ is the transitional time constant.

The roots of the characteristic equation are:

$$\begin{array}{c} {\left(\tau s\right)}^{n}=-1=e^{j(\pi +2k\pi )} \end{array}$$ (22)

for *k* integer and

$$\begin{array}{c} s=\frac{1}{\tau }e^{\pm j\frac{\pi }{n}} \end{array}$$ (23)

These roots will be complex conjugated and determine the oscillatory nodes of the relaxation. The time constant depends on the mass *M* and when it varies, the roots move along a straight line with an angle *ϕ* determined exclusively by the fractional order *n*. Since this *n* does *not* depend on mass, the damping properties will not vary with the mass, but with the structural properties of the porous material—in our case the alveolar structure. The damping if calculated as:

$$\begin{array}{c} \zeta =\cos\left(\phi \right)=-\cos\frac{\pi }{n} \end{array}$$ (24)

whereas the natural frequency of such a structure is given by

$$\begin{array}{c} \omega_{n}=\frac{1}{\tau } \end{array}$$ (25)

In conclusion, we have shown that properties of a single alveolus, as well as those of a network of alveoli may be naturally characterized by fractional order impedance models.

### 2.3 Structural changes in COPD

Chronic obstructive pulmonary disease (COPD) is often paired with emphysema and referred to as small airway disease [15, 31]. It features a prolonged time constant for lung deflation, due to increased resistance of the small conducting airways and increased compliance as a result of emphysematous destruction. The latter implies breaking the alveolar walls and disruption of the adjacent connective walls, inflammation and tissue mass enlargement. Density of the lung is decreased, with airspace enlargement and CT scans have shown various degrees of heterogeneity in density of porous tissue with various degrees of obstruction [32].

**Fig 3:**
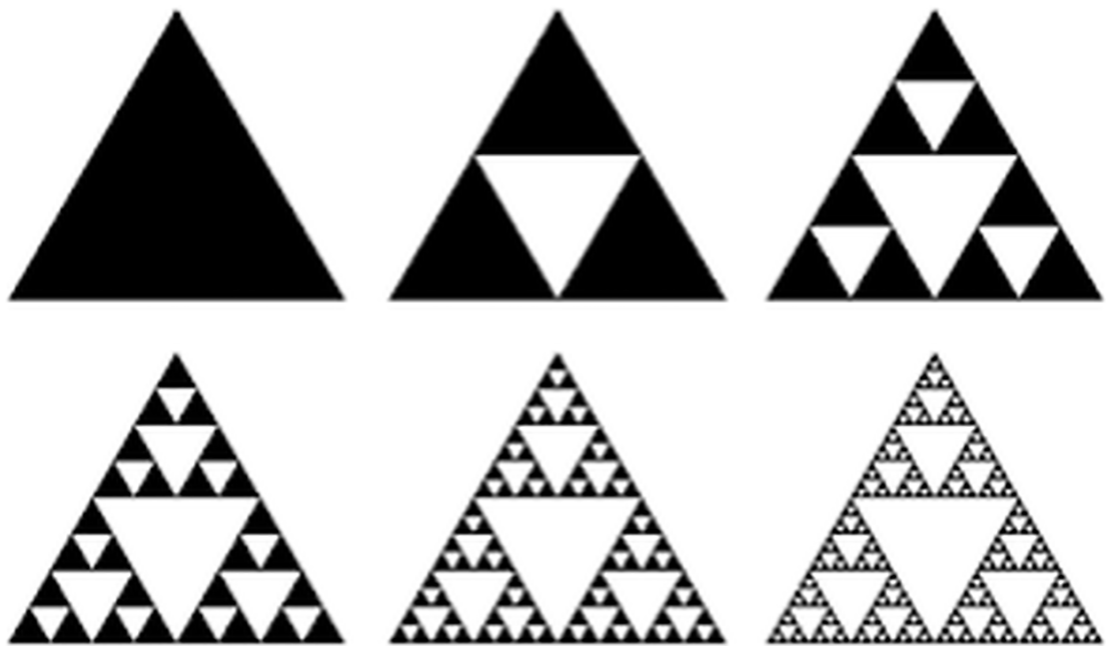
Classical Sierpinski triangle (left) and Sierpinski-like arrangement of alveolar areas with various degrees of density.

Using a gross analogy to the Sierpinski triangle of various densities, as illustrated in Fig 3, we can enumerate the following changes in our impedance model as a result of airway remodelling in COPD:

- the *R* and *C* parameters, along with their corresponding recurrent ratios, will vary in different respiratory zones;
- the alveolar changes will affect the fractional order term value *n*;
- within respiratory zones, different such fractional order term values will exist;
- the porous structure of the network of alveoli will then contain multi-fractal spacial distribution and thus multi-fractal dynamics.

Fractal and multi-fractal analysis has been given lots of attention in medical applications [10]. Specifically for lung applications, dissimilarity principle has been applied to classify lung parenchyma based on image processing techniques [33], as well as pulmonary emphysema [34]. Airway remodelling has been correlated to fractal dimension in asthma [35], providing insight into space filling mechanism. Pulmonary embolism has been detected using multifractal analysis from lung scans and provided useful classification tool [9].

It is interesting to provide a correlation between changes in COPD at the alveolar level and fractional order impedance values evaluated in various degrees of obstruction. As illustrated in Fig 3, as COPD progresses, changes in the *R*, *C* parameters and structure of the alveolar network will affect the impedance and thus the lung function will change.

As the ratios of resistance and capacitance increase with various degrees of COPD obstruction (i.e. classified as GOLD2, GOLD3 and GOLD4, from mild to severe degree of obstruction), the fractional order term value *n* will vary. If the relative increase is the same, the value will remain unchanged. If resistance has absolute variance higher than capacitance, then the values of *n* will increase. Similarly, if capacitance has absolute variance higher than resistance, then the values of *n* will decrease.

The implications for damping are complex. Due to relative increase in compliance as a result of more empty spaces in the alveolar structure, damping will apparently decrease, but due to increased tissue density, the overall damping is increasing. Notice that if alveolar surface is decreased with pathology, the time constant defined in previous section for the network will also increase. This is in line with the pathology of chronic obstructive pulmonary disease (COPD) which has long deflation periods. Typically, the natural frequency in COPD is usually increased, implying that as the time constant is increased, the term in *n* from Eq (25) has to increase faster than the term in *τ*. In conclusion, local structural changes are more important than the network spacial distribution in terms of impedance.

### 2.4 Patients

The study includes 43 COPD diagnosed patients (≥ 60 years) who came for periodic evaluation of their lung function at Ghent University Hospital, Belgium. The study group included: 18 subjects diagnosed with COPD-GOLD II; 15 subjects diagnosed with COPD-GOLD III and 10 subjects diagnosed with COPD-GOLD IV. Biometric and spirometric variables are listed in Table 1. Written informed consent was obtained from all participants. This study and the consent procedure was approved by the local ethical committee of Ghent University Hospital, Ethical advice number B670201111936.

**Table 1:** Biometric characteristics data for the subjects included in the study.

-	COPD-GOLD II	COPD-GOLD III	COPD-GOLD IV
# Patients	18	15	10
Age (yrs)	71 ±9.4	77±5.7	68±3.5
Height (m)	1.62±0.07	1.7 ±0.087	1.66 ±0.035
Weight (kg)	70.4 ±10.59	78.5 ±10.66	75.4 ±13.64

### 2.5 Lung function device

**Fig 4:**
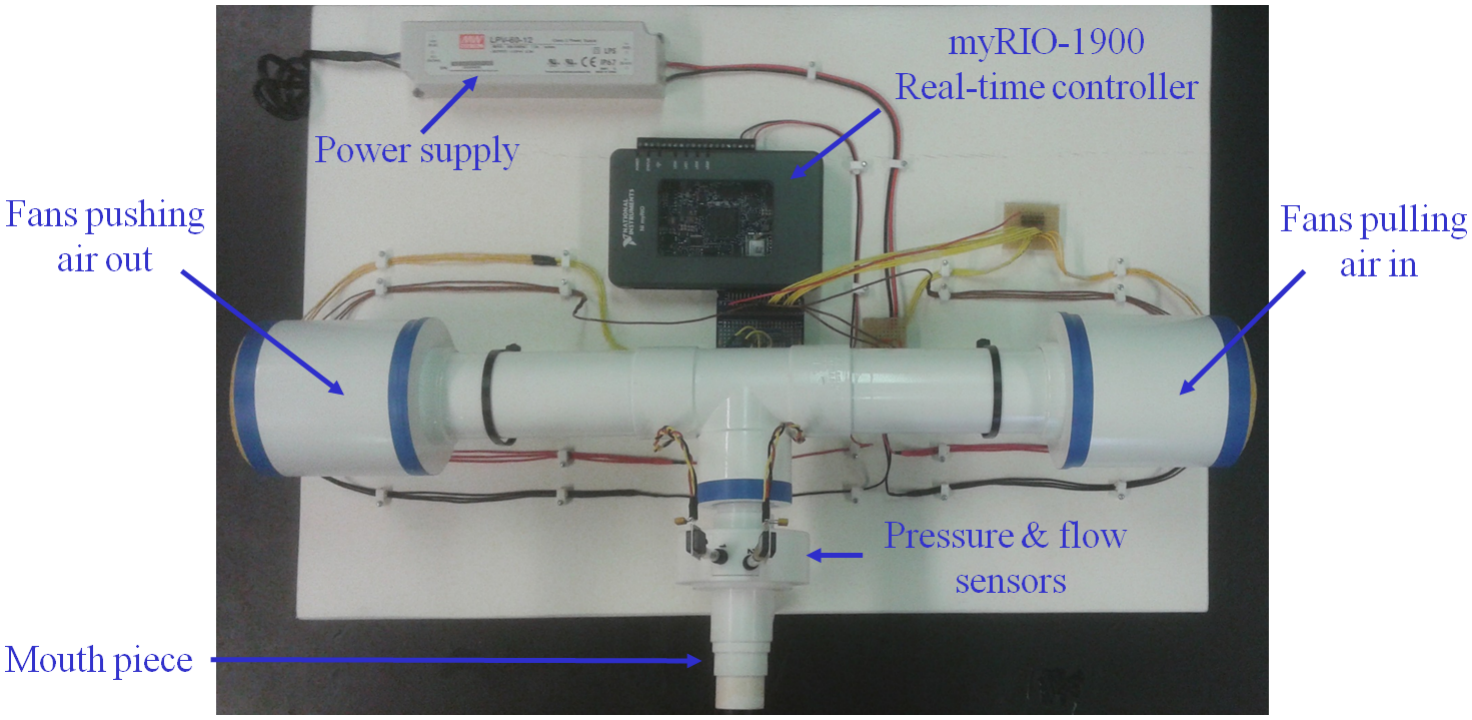
Photo of the measurement device and related instrumentation.

The device presented in this paper and used for measurements is a third generation of the prototype described in [36–38]. The system consist in a group of fans located on each extreme of the principal pipe (see Fig 4).

One group pushes the air into the tube, while the group on the other side of the tube, extracts the air. The controlled difference in speed between the two groups will generate a controllable pressure inside the pipe. The pipe has 2 inches diameter and has been filled with tubes with smaller diameter (straws) in order to preserve laminar flow. Despite the careful design, nonlinear effects are not completely avoided and their contribution may be accounted for. The end part of the device is formed a pneumotachograph and two pressure sensors. A mouthpiece is mounted on the device for every patient tested (single-use). Since the main goal is to design a suitable optimization technique in order to improve the signal-to-noise-ratio in the signal of interest, efforts have been made to develop a device able to perform low frequency measurements. By measuring at frequencies close to the breathing frequency of the patient, important distortions are present in the system coming from the interference of the breathing signal with the desired excitation signal (typically an optimized multisine). One of the approaches was to introduce open loop feedforward compensation (FF) and closed loop feedback compensation (FB). This has been achieved with online estimation of breathing period for FF and Proportional-Integral-Derivative controller design for FB.

## 3 Results and discussion

### 3.1 Analysis of the model in simulation

**Fig 5:**
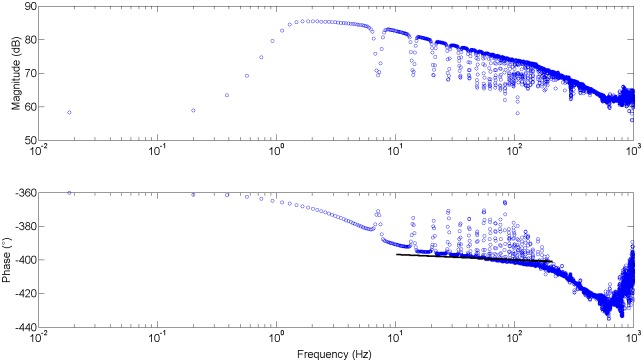
Explicit impedance for individual alveolus simulated with healthy lung parameters. Black thick line denotes the constant phase of the lumped impedance.

Fig 5 depicts the impedance of an isolated alveolus with particular lung tissue parameters.

These parameters are given below, with units as given above [39–41]:

$$\begin{array}{c} \rho =350;c=5000;\lambda =0.05; \end{array}$$ (26)

**Fig 6:**
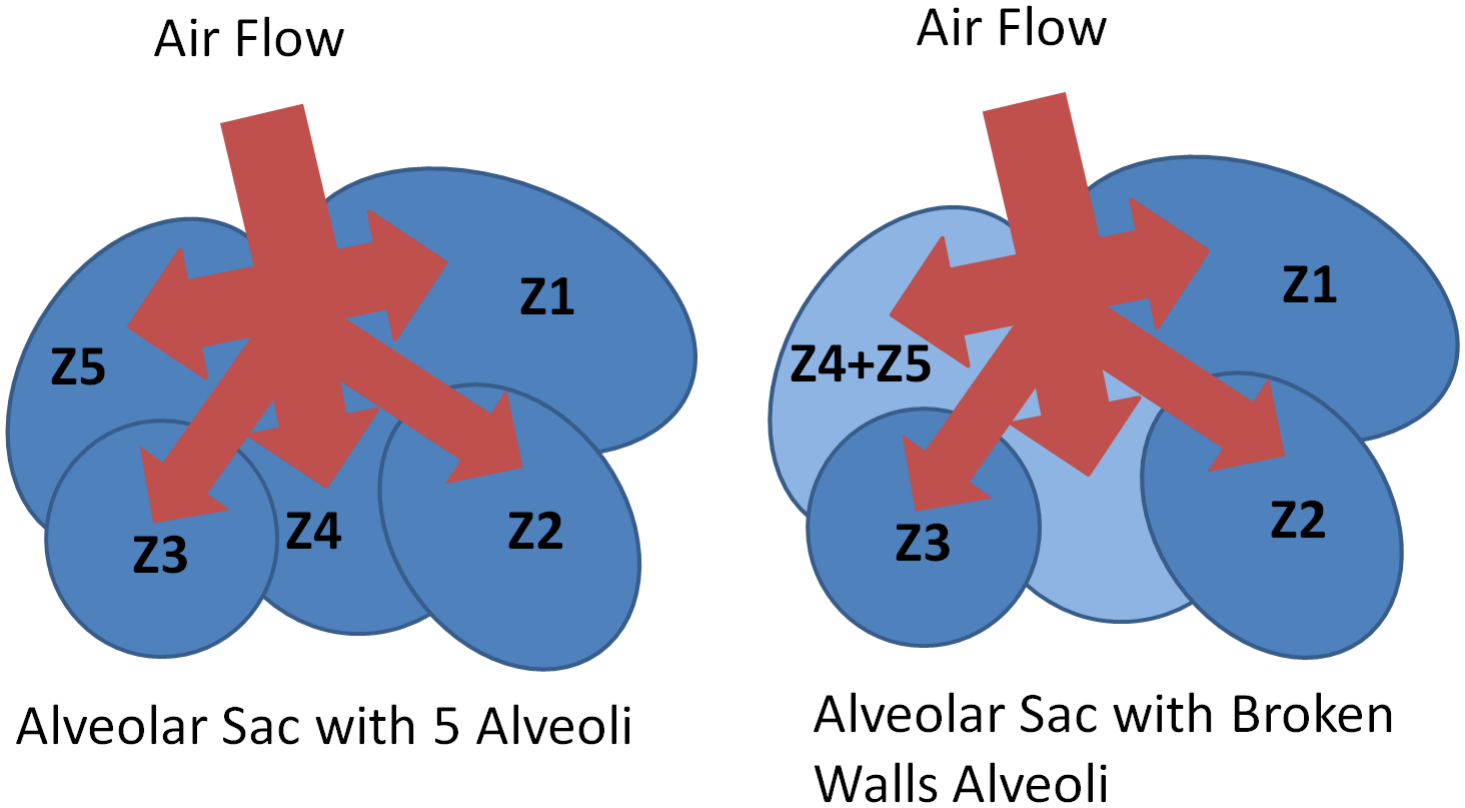
Schematic representation of an alveolar with 5 alveoli (left) and with broken alveoli (right).

It can be observed that the lumped impedance follows closely the explicit impedance in a limited frequency range. This interval will depend on the parameters Eq (26). Notice that changes with disease at alveolar level will affect the cell elements Eq (10), consequently will change the lumped impedance form and affect the fractional order operator. Using the impedance of an alveolus, we can now construct a network of alveoli. The origin of this impedance is not essential, as long as its form has the term in fractional order to help us understand changes with remodelling. Supposing that the flow is divided equally through the 5 alveolar sacs as in Fig 6, then all impedances are equal and receive the air-flow in parallel.

This allows us to write the following admittance equation:

$$\begin{array}{c} Yt=Y1+Y2+Y3+Y4+Y5 \end{array}$$ (27)

with *Yt* = 1/*Zt* the total admittance and *Y*_*i*_ the admittances of each alveoli, with the form from Eq (14), with fractional order value *n* = 0.5. The total admittance will not change in phase, only in amplitude, dependent on the amount of alveoli we take into calculus of Eq (27). In pathology, as explained before, the mechanical properties are altered as a result of remodelling and lead to different fractional order values. Structure of alveolar sacs also modifies, leading to various consistencies of network of alveoli, as conceptually illustrated in Fig 3. So if we consider that we have the same admittance form as in Eq (27), but with the following elements:

$$\begin{array}{c} \begin{array}{c} Y1=Y2=1/Zs\\ Y3=Y4=Y5=1/Zs”\\ Zs(n=0.5)\;\;and\;\;Zs”=Zs(n=0.3) \end{array} \end{array}$$ (28)

where *Zs* has the same form and values as in Eq (14) except the fractional order *n*. The phase will be altered, varying between −0.3 * 90° and −0.5 * 90°.

In COPD, we also have the breaking of alveolar walls, creating larger spaces. This will induce a different form of the admittance, as:

$$\begin{array}{c} Yt=Y1+Y2+Y3+\frac{1}{Z4+Z5} \end{array}$$ (29)

with the same definitions as Eq (14) and *n* = 0.5 for *i* = 1, 2, 3 and *n* = 0.3 for *i* = 4, 5. This will also affect the phase of the total admittance, but the limit values will be the same as those from Eq (28). This mathematical framework provides the necessary support for the multi-fractal dynamics exhibited by the lung parenchyma and much discussed in literature under the resulting property of self-organized critically system Phase dynamics and thus also phase transitions are direct result of remodelling effects as a self-defence mechanism of the respiratory system towards disease.

**Fig 7:**
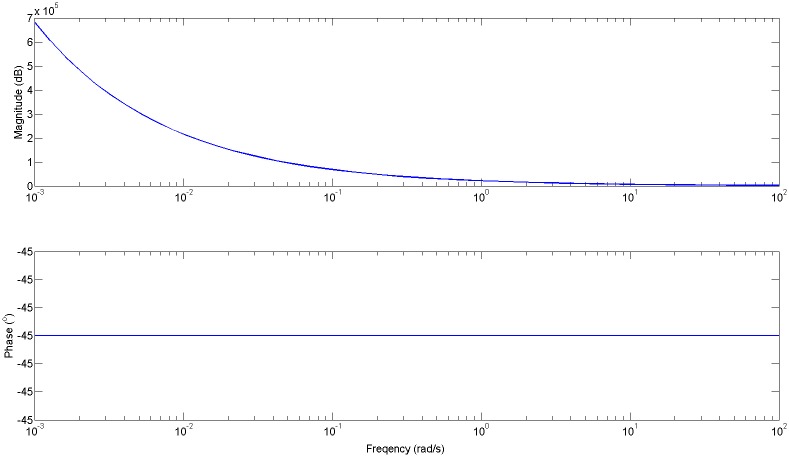
Impedance (*Zt* = 1/*Yt*) for all alveoli equal and *n* = 0.5 from Eq (27).

**Fig 8:**
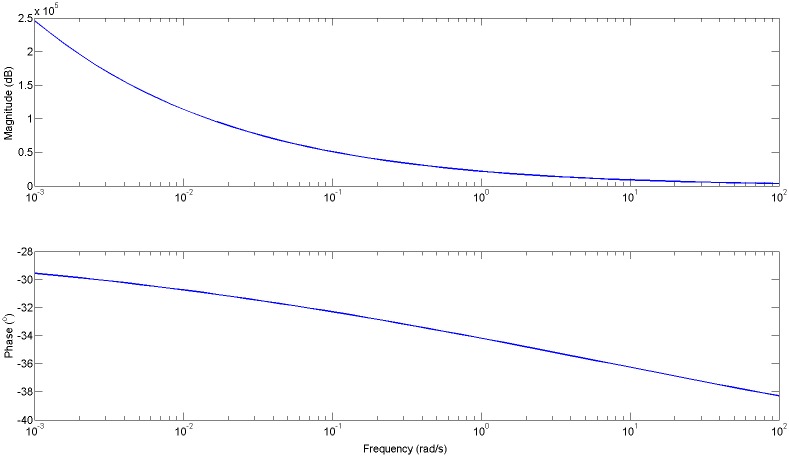
Impedance (*Zt* = 1/*Yt*) for mixed types of alveoli with impedances varying fractional orders *n* = 0.3, *n* = 0.5, as given in Eq (28).

The result for the impedance obtained with Eq (27) is depicted in Fig 7 and from Eq (28) is depicted in Fig 8.

It can be observed that the limit phase values are close to the expected values related from the fractional orders (−30°, −45°).

**Fig 9:**
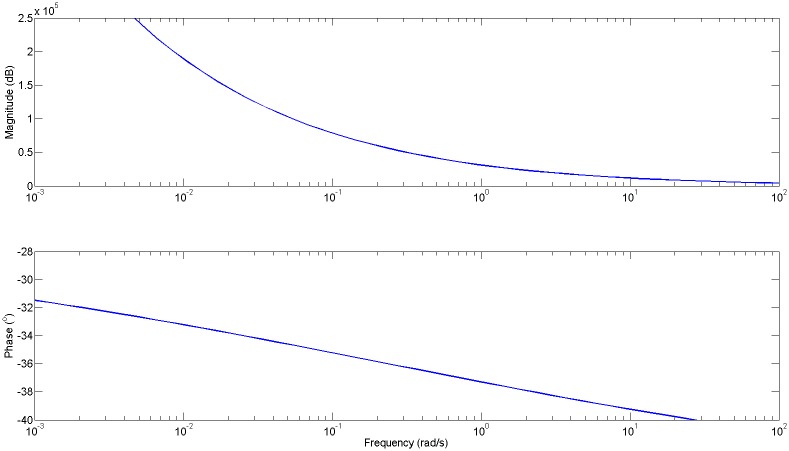
Impedance (*Zt* = 1/*Yt*) for broken alveolar walls and mixed types of alveoli with impedances varying fractional orders *n* = 0.3, *n* = 0.5, as given in Eq (29). The phase is again affected, compared to the result from Fig 8.

Finally, the result of the impedance with broken alveolar walls, as defined in Eq (29) is given in Fig 9.

As a general remark about all these simulation results, the magnitude values are unrealistically high. In reality, the very high alveolar surface (2^24^ alveolar sacs according to [17]) will reduce significantly the magnitude for a single sac. Here we used generic values to illustrate the effects of various changes occurring in such structures with pathology.

The simulation studies performed here suggest that the convergence of the fractional order in model from Eq (7) has a structural origin and variations are directly related to structural changes. To our knowledge, such an analysis has not yet been reported in specialised literature.

**Fig 10:**
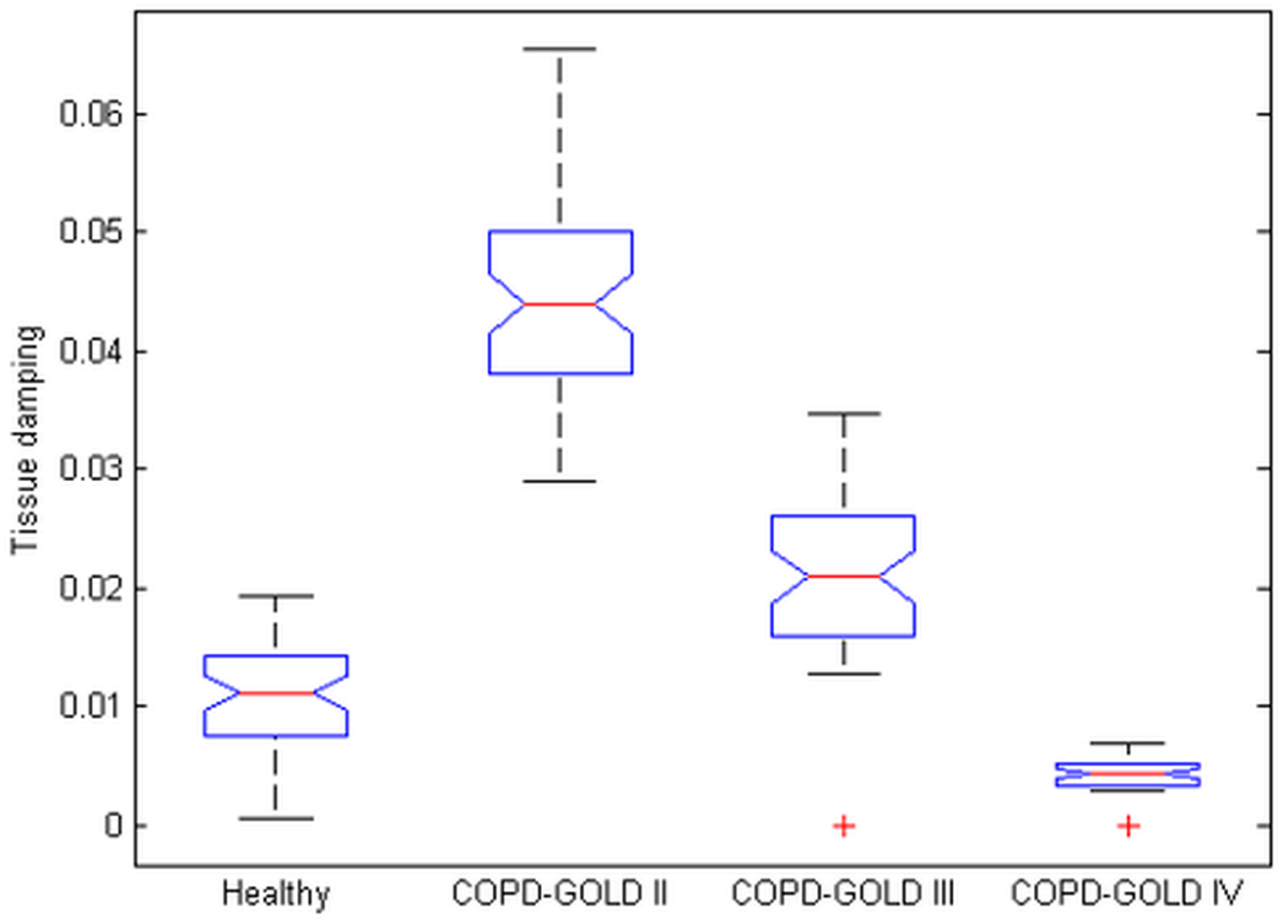
Simulated tissue damping (*G*_*r*_) in: 1—healthy, 2—COPD-GOLD II, 3—COPD-GOLD III and 4-COPD-GOLD IV (chronic obstructive pulmonary disease stage II, III and IV).

Changes in gas exchange area paired with disease evolution are related to the mass of air which can be involved in the ventilation process. This implies that the term in Eq (21) has a time constant which is varying with the relative ratio between gas exchange surface and air mass. The damping factor of such a system is independent on this term, but the natural frequency is inversely related to it via relation Eq (25). The natural frequency will increase for same surface with lower air mass, as expected in COPD patients. This is then always reflected by the balance of inertial and capacitive properties in these patients which tend to be equal at frequencies around 15 Hz and higher (in healthy, the resonant frequency is around 8Hz). In terms of respiratory impedance, the resonant frequency is where the negative part of the imaginary part of impedance (reactance) is equal to the positive part (i.e. crosses the zero line) [13, 16]. The damping factor is a parameter reflecting the capacity of the material for energy absorption. In the case of lung tissue damping is mostly characterize by viscoelasticity. The increased lung elastance in COPD due to more empty spaces in the alveolar structure will result in higher values of tissue damping than in healthy patients. The model from Eq (21) has been used to mimic healthy and COPD patient by changing morphological parameter values in Eqs (12) and (13). This impedance was then fitted to the model in Eq (7) to extract parameters from Eq (8). The results are given in Fig 10 for tissue damping (*G*_*r*_).

It can be observed that as damping is increased, in COPD it relaxes as the alveolar walls are broken with COPD progress.

### 3.2 Experimental data

We do not have the possibility to measure non-invasively the impedance of the alveolar network. Instead, we employ the forced oscillation technique, a well-known lung function test broadly used to assess mechanical properties in lungs [16].

**Fig 11:**
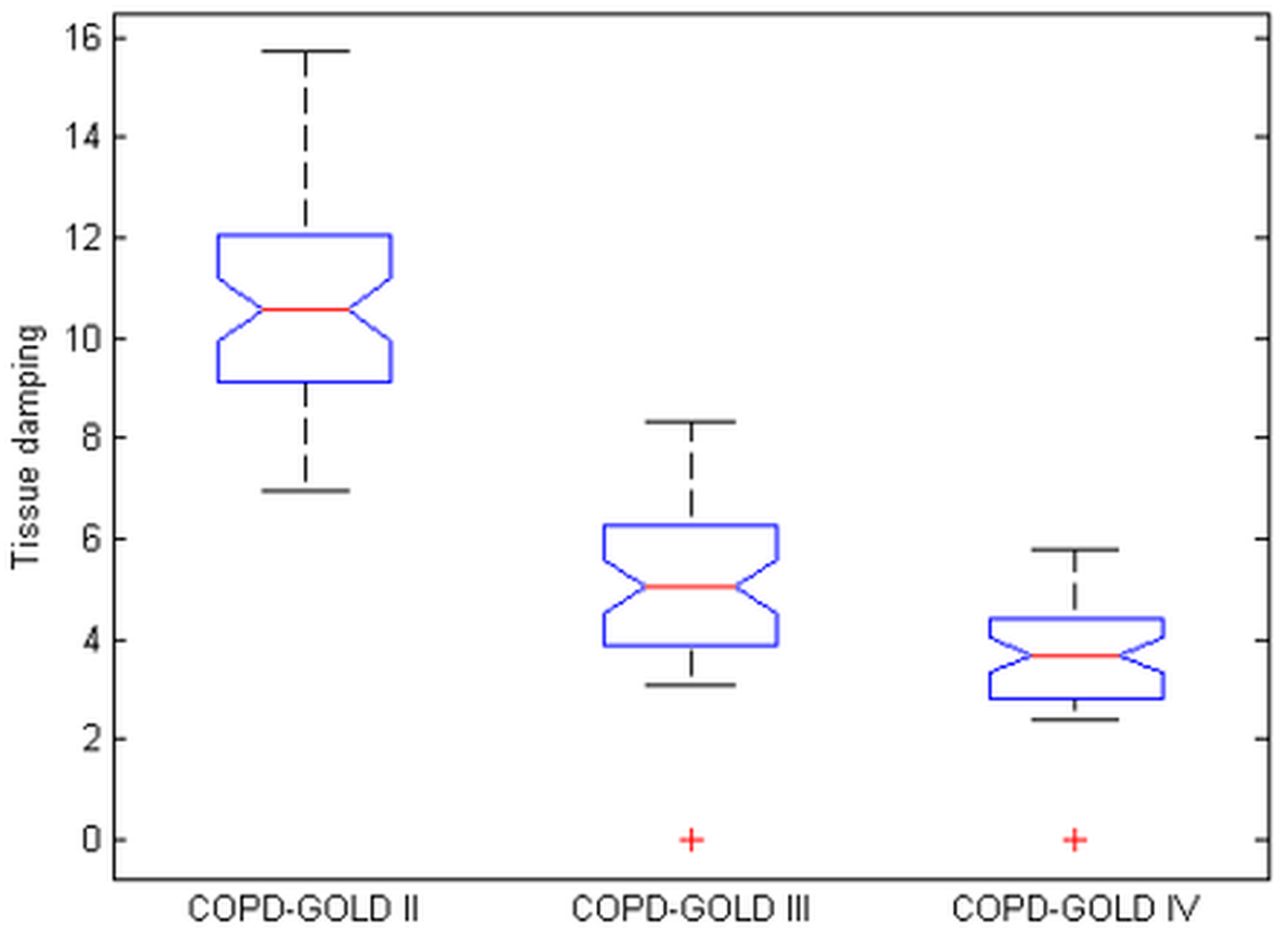
Tissue damping (*G*_*r*_) in: 1—COPD-GOLD II, 2—COPD-GOLD III and 3—COPD-GOLD IV (chronic obstructive pulmonary disease stage II, III and IV).

**Fig 12:**
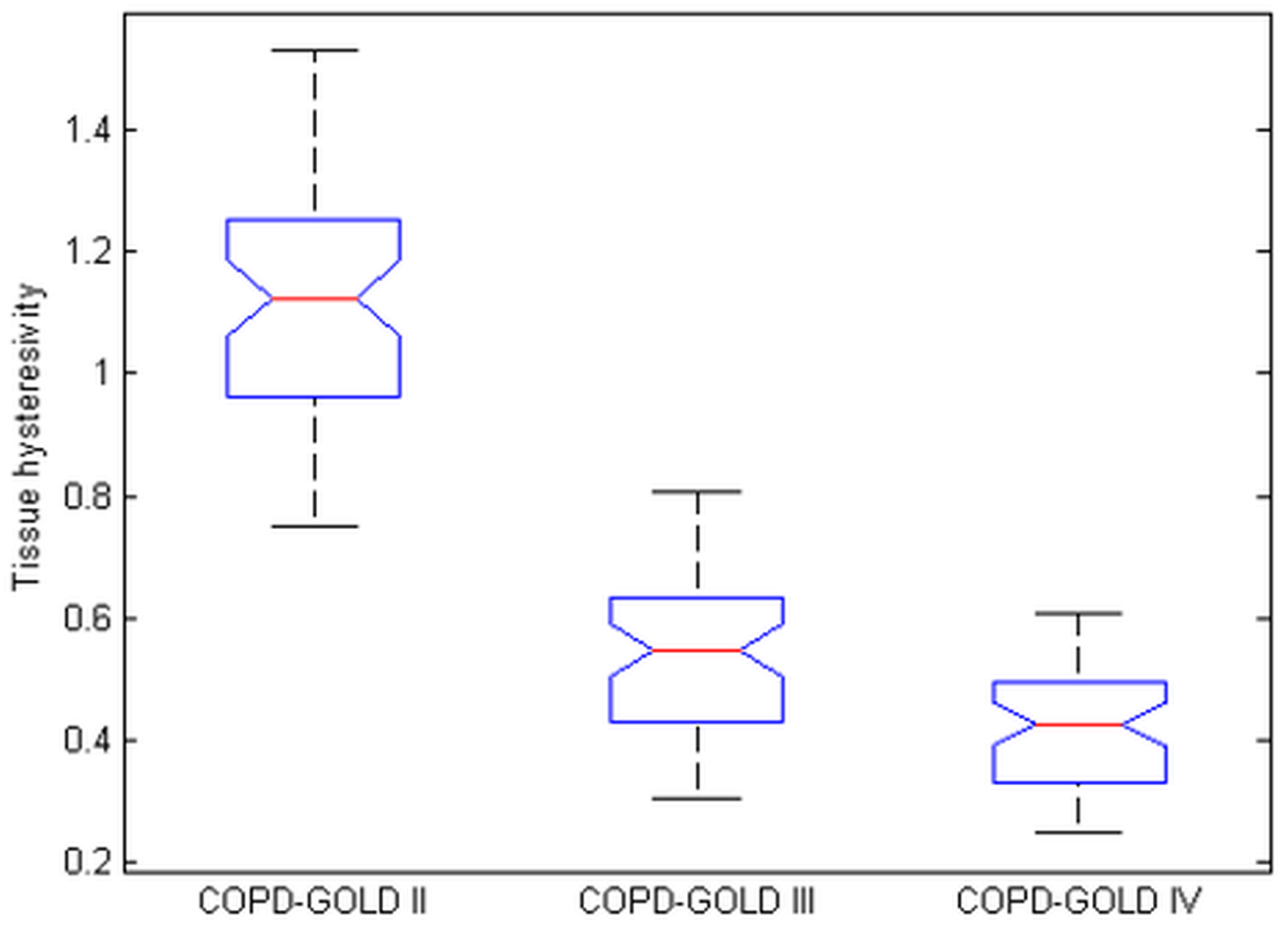
Tissue hysteresivity (*η*_*r*_) in 1—COPD-GOLD II, 2—COPD-GOLD III and 3-COPD-GOLD IV (chronic obstructive pulmonary disease stage II, III and IV).

Figs 11 and 12 depict the boxplots for the tissue damping *G*_*r*_ and tissue hysteresivity *η*_*r*_. Statistical analysis has been performed to identify if a significant difference between the group exists. Anova tests have been applied to the data and it has been observed a significant difference in tissue damping between the three groups: i.e. between: COPD-GOLD II and COPD-GOLD III; COPD-GOLD II and COPD-GOLD IV; COPD-GOLD III and COPD-GOLD IV. The p values identified for this case is lower than 0.01. Same analysis has been performed also for tissue hysteresivity and a p value lower than 0.05 has been identified.

The damping factor is a material parameter reflecting the capacity for energy absorption. In materials similar to polymers, as lung tissue properties are very much alike polymers, damping is mostly caused by viscoelasticity, i.e. the strain response lagging behind the applied stresses [4, 5]. The loss of lung parenchyma (empty spaced lung), consisting of collagen and elastin, both of which are responsible for characterizing lung elasticity, is the leading cause of increased elastance in COPD. Damping will increase due to increased tissue density in places where alveolar walls are not broken.

**Fig 13:**
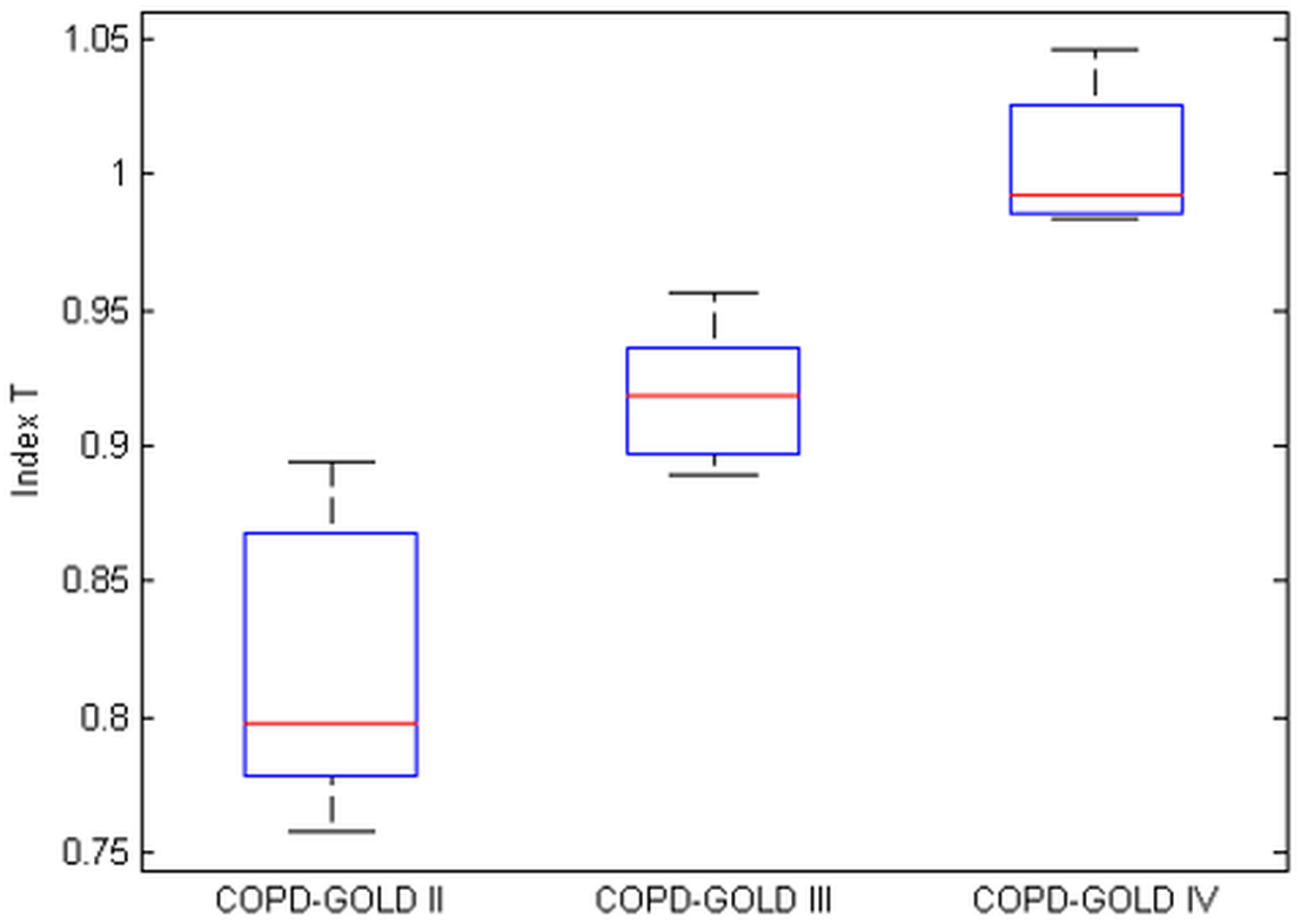
Boxplot for the non-linear distortions in COPD-GOLD II, COPD-GOLD III and COPD-GOLD IV diagnosed groups. There is a significant difference between groups.

Since pathology of COPD involves significant variations between inspiratory and expiratory air-flow, an increase in the hysteresivity coefficient *η*_*r*_ reflects increased inhomogeneities and structural changes in the lungs. In emphysematous lung, the caliber of small airways changes less than in the normal lung (defining compliant properties) and peripheral airway resistance may increase with increasing lung volume. Based on our proposed derivations to obtain Eqs (5) and (21) the model from Eq (7) has a strong theoretical basis linked to i) mechanical (resistance, elastance) properties of lung parenchyma, ii) structural layout (recurrent properties) and iii) tissue density (porous character). Alterations in these properties have an effect of the overall impedance parameters and these can now be linked to changes in small airways and alveolar levels. This in turn affects the degree of non-linearity in the tissue dynamics. The non-linear index T has been calculated for each group and the results obtained are presented in Fig 13.

It can be noticed that there is a significant difference between the groups. From the results obtained we may conclude that the non-linear index T increases as COPD disease advances which is in line with prior rationale. Statistical analysis for the the non-linear T-index in the measured group has been performed and the results obtained are reported in Table 2.

**Table 2:** Confidence intervals for the calculated non-linear distortions index T in the measured groups. Std denotes standard deviation.

Group	Min	Max	Mean	Std
COPD-GOLD II	0.7579	0.9840	0.8228	0.0488
COPD-GOLD III	0.8893	0.9568	0.9205	0.0229
COPD-GOLD IV	0.9836	1.0459	1.0019	0.0214

**Fig 14:**
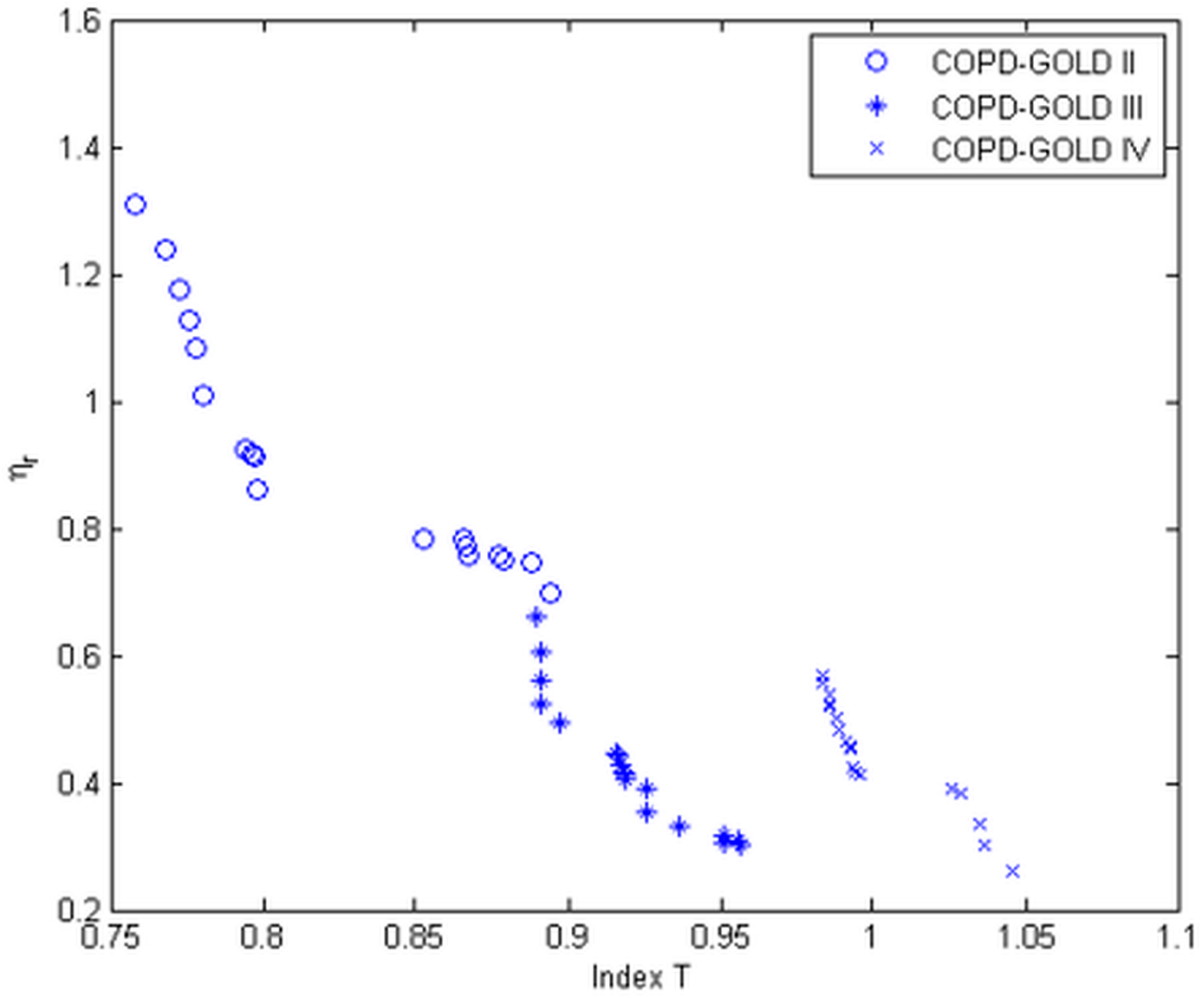
Relation between non-linear index and the heterogeneity factor (*η*_*n*_) is the evaluated COPD groups. ‘o’ denote COPD-GOLD II, ‘*’ denote COPD-GOLD III and ‘x’ denote COPD-GOLD IV.

In Fig 14 the evolution of the hysteresivity factor as a function of the non-linear index is presented.

The results obtained indicate that as COPD continues to deteriorate the alveolar walls the heterogeneity of the lungs will decrease as larger spaces occurs. However, the effects from changes at macromolecular level contribute to increased non-linear dynamics. The results indicate there exist a correlation between the non-linear index T and the heterogeneity factor (i.e. *T* increases ad the COPD disease advances). The added value of the T index is that it can be extracted directly from the measured signals without the necessity of Eq (7).

## 4 Conclusions

This paper presented the available tools emerging from fractional calculus to model changes in chronic obstructive pulmonary disease (COPD) and to understand their effect on model parameters values. The theoretical basis helps in understanding the interplay between all these structural and dynamical changes. The results obtained indicate that there is a direct relation between the non-linear index T and the heterogeneity in COPD lungs. A limitation of this study is that effects of decrease in vascularisation associated with broken alveolar walls are not investigated. This implies an augmentation of the model with complex mathematical description of perfusion and diffusion phenomena. However, However, these are pertinent next steps in further developing the mathematical of fundaments respiratory mechanics.

*P*—respiratory pressure [*kPa*]

*Q*—airflow, blood flow [*m*^3^/*s*]

*C*—elastance [*L*/*kPa*]

*V*—air volume [*L*]

*R*—resistance [*kPa* *s*/*L*]

*P*_0_—pressure at the end expiration [*kPa*]

*I*—inertia [*KPas*^2^/*L*]

*r*—wall radius [*cm*]

*h*—wall thickness [*cm*]

*μ*—viscosity [*kg* *m* *s*]

*ρ*—density [*kg*/*m*^3^]

*l*—length of the airway tube [*cm*]

*M*_1_—modulus of Bessel function

*R*_*e*1_—resistance in the first airway [Ω]

*C*_*e*1_—compliance in the first airway [*F*]

λ—ratio of resistances/total levels

*χ*—ratio of compliances/total levels

*s*—Laplace operator

*K*(λ, *X*)—gain factor

*n*—fractional order operator

*Z*_*N*_(*s*)—impedance

*M*—air mass [*g*]

*v*—air velocity [*m*/*s*]

*F*—membrane force [*N*]

*G*—tissue damping [*kPas*^1−*β*^/L]

*H*—tissue elastance [*kPas*^1−*β*^/L]

*r*_1_—inner radius of the cell [cm]

*r*_2_—outer radius of the cell [cm]

*R*_*i*_—resistance of cell *i*

*C*_*i*_—compliance of cell *i*zz *N*—total number of cells

*S*_*i*_—surface of cell *i*

*S*—alveolar surface

*m*—fractional order value dependent on the ratios of the alveolar parameters

*ζ*—damping factor

*ω*_*n*_—natural frequency

*ω*_*k*_—frequency line [rad/s]

*T*_*i*_—temperature [°*C*]

## Supporting information

S1 Table Identified model parameters for each analyzed group. *Tindex* denotes the nonlinear index, *η* denotes the hysteresivity coefficient and *G* denotes the tissue damping.
(XLSX)
